# Should We Monitor ScVO_2_ in Critically Ill Patients?

**DOI:** 10.1155/2012/370697

**Published:** 2011-09-21

**Authors:** Sophie Nebout, Romain Pirracchio

**Affiliations:** ^1^Department of Anesthesiology and Critical Care Medicine, Hospital Lariboisière 75475, Paris, France; ^2^Department of Anesthesiology and Critical Care Medicine, Hôspital Européen Georges Pompidou, Université Paris V Descartes, Sorbonne Paris Cité, 20 Rue Leblanc, 75015 Paris, France

## Abstract

Hemodynamic monitoring has become a real challenge in the intensive care unit. As an integrative parameter for oxygen supply/demand, venous oxygen saturation (SvO_2_) provided by pulmonary artery catheterization is one of the most popular parameters to assess the adequacy of cardiac output. However, technical limitations and potential iatrogenic complications constitute important limits for a widespread use. Regular central venous catheters coupled with a fiberoptic lumen for central venous oxygen saturation (ScvO_2_) monitoring have been proposed as a surrogate for SvO_2_ monitoring. The purpose of the present article is to review the physiological backgrounds of circulation, the pathophysiology of circulatory failure and subsequent venous oxygen saturation alterations, and finally the merits and the limits of the use of ScvO_2_ in different clinical situations.

## 1. Introduction

Hemodynamic monitoring has become a common practice in the intensive care unit. Besides blood pressure measurement, most industrial efforts have concentrated on providing devices for cardiac output monitoring. However, adequate adaptation of these macrohemodynamic parameters is somehow challenging. Indeed, as cardiac output is an adaptive parameter, it is always difficult to judge whether a *given value* at a *given time* for a *given patient* is appropriate or not. Similarly, which value should be considered an appropriate goal for blood pressure, considering regional perfusion specificities (e.g., autoregulation or flow/pressure dependency), patient's age, history of hypertension, and so on. Therefore, considering that O_2_ supply to the tissue is the basic objective, intensivists have been trying to find out an integrative parameter that would be more suitable to globally assess hemodynamic status of their patients. As a surrogate for evaluating O_2_ demand/supply adequacy, central oxygen venous saturation (ScvO_2_) has become a popular parameter. As explained for the dummies, oxygen venous saturation is interpreted as a bank statement at the end of the month: “if the balance is negative, you can consider two explanations: you spend too much money or you earn not enough.” The aim of the present paper is precisely to critically analyze the physiological basements for such an interpretation, the data that support its use in clinical practice, and finally the limits that should be kept in mind while using such a parameter at the bedside.

## 2. Physiological Background

### 2.1. Normal Circulation Physiology

One of the main goals of blood circulation is to ensure oxygen supply to organs and tissues. The determinants of arterial oxygen delivery (DO_2_) are

- cardiac output (CO);
- arterial content in oxygen (CaO_2_).

The arterial content in oxygen has 2 components.

1. The main component is oxygen bound to hemoglobin (Hb).
2. The secondary component is dissolved oxygen.

The first one depends on hemoglobin concentration, hemoglobin affinity for oxygen (which varies for Hb isotypes and with environmental conditions such as temperature, pH, or 2.3 DPG concentrations), and, therefore, Hb oxygen saturation. The second component depends on arterial partial pressure of oxygen (PaO_2_) and is considered as negligible because of the very solubility coefficient of oxygen in plasma. It is then possible to set the equations:

- CaO_2_ = (Hb × 1.34 × SaO_2_) + (0.003 × PaO_2_),
- DO_2_ = CO × CaO_2_.

By ignoring the dissolved oxygen component, we get

- DO_2_ = CO × 1,34 × SaO_2_.

Arterial blood is then deoxygenated in tissues. Tissue oxygen extraction depends on their demand but also on their ability for oxygen extraction. Therefore, after peripheral oxygen extraction, venous oxygen content depends on arterial content and tissue oxygen extraction.

### 2.2. Pathophysiology of Circulatory Failure

Shock is one of the leading causes of admission in the intensive care unit. It is usually defined as a mean arterial pressure (MAP) <60 mmHg or a systolic arterial pressure (SAP) <90 mmHh, or a decrease in SAP greater than 40 mmHg as compared to the usual SAP [1]. For many years, hemodynamic management has focused on “macrocirculatory” parameters such as blood pressure or cardiac output. Though the magnitude of macrocirculatory disorders is well known to be related to prognosis [2], its optimization seems mandatory [3] but insufficient [4]. Indeed, in septic shock patients, Sakr et al. observed that after 24 h hours of intensive care, the values of MAP, cardiac index (CI), and central venous pressure (CVP) did not discriminate survivors from nonsurvivors. 

**Figure 1:**
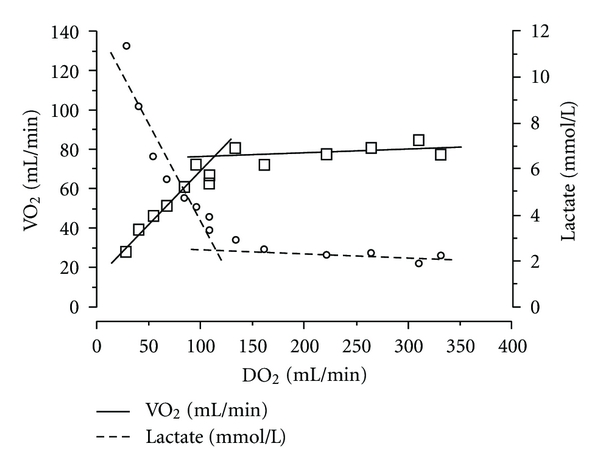
Evolution of oxygen consumption when oxygen delivery decreases. From Vincent and De Backer [5] with permission. Note the presence of a DO_2_ threshold located at approximately 100 mL/min. Below this value, oxygen consumption begins to fall and lactate concentration increases, indicating a switch from aerobic to anaerobic metabolism.

Hence, shock can be defined as a *macrohemodynamic* instability leading to an inappropriate oxygen supply/demand balance. Schematically, as represented in Figure 1, any fall in DO_2_ is initially compensated by an increase in tissue oxygen extraction (EO_2_), explaining that tissue VO_2_ is initially maintained. However, when tissue oxygen extraction capacity is overtaken, oxygen consumption begins to fall and lactate concentration increases, indicating a switch of the cellular metabolism from aerobic glycolysis to cytoplasmic anaerobic glycolysis. This threshold immediately precedes the onset of clinical organ failures.

Considering such a pathophysiological scheme, hemodynamic support in shock should aim at correcting macrocirculatory but also microcirculatory parameters in order to avoid any local fall in O_2_ supply below this crucial threshold. Therefore, a parameter such as venous oxygen saturation (SvO_2_), that should reflect the inadequacy in oxygen supply, might be of great help.

### 2.3. Physiological Determinants for Venous Oxygen Saturation

Oxygen extraction in the tissues can be mathematically defined as follows:

- EO_2_ = CO × (CaO_2_ − CvO_2_),
- EO_2_ = VO_2_/DO_2_,

with CvO_2_ being venous oxygen content and VO_2_ being oxygen consumption.

Then, venous oxygen saturation can then be calculated using the following formula:

- SvO_2_ = SaO_2_ − (VO_2_/(CO × Hb × 1.34)).

Hence, any decrease in venous oxygen saturation should be explained by

- a decrease in SaO_2_;
- a decrease in cardiac output;
- a decrease in hemoglobin level;
- an increase of oxygen consumption (VO_2_).

Thus, providing that SaO_2_, oxygen consumption, and haemoglobin level are in normal ranges, SvO_2_ can be used as a surrogate for cardiac output.

Likewise, if

- EO_2_ = CO × (CaO_2_ − CvO_2_),
- EO_2_ = VO_2_/DO_2_,

then,

- EO_2_ = (SaO_2_ − SvO_2_)/SaO_2_.

Consequently, when SaO_2_ = 100%, then EO_2_ = 1 − SvO_2_ and SvO_2_ = 1 − EO_2_.

Then, SvO_2_ is also a good surrogate for EO_2_.

In shock, decrease in tissue oxygen supply is mostly related to a decrease in tissue blood flow, would it be relative (as in distributive shocks) or real (as in hemorrhagic shock). The first recommended measure in international guidelines for shock resuscitation consists in optimizing cardiac output by repeated fluid challenges [6], in order to correct oxygen supply/demand imbalance. In this aspect, SvO_2_ measurements could help guiding fluid challenges in shock patients.

## 3. SvO_**2**_—ScvO_**2**_: Is It the Same?

The reference technique to assess the adequacy of oxygen supply is the mixed venous oxygen saturation (SvO_2_), provided by pulmonary artery catheter (a.k.a. Swan-Ganz catheter) [7]. However, limitations related to difficulties of insertion and placement, but also to potential complications related with such a catheter, lead to a substantial decrease in its use. In the meantime, industrials have developed regular central venous catheter coupled with a fiberoptic lumen for continuous haemoglobin saturation monitoring. Placed through a jugular of a subclavian vein, at the confluent of the superior vena cava and the right auricle, such catheters actually monitor the central venous oxygen saturation (ScvO_2_) [8]. 

However, one should ask whether SvO_2_ and ScvO_2_ provide the same information. Actually, taken in pulmonary artery, SvO_2_ is a surrogate for global tissue oxygenation, whereas ScvO_2_ essentially reflects the oxygenation of the upper part of the body (head, neck upper limbs, and upper part of the trunk) and of a lower proportion of the lower part of the body (lower part of the trunk and lower limbs), depending on the exact position of the catheter's extremity. Anyhow, ScvO_2_ does not include venous blood coming from coronary sinus commonly located in the right auricle. Thus, taken at the confluent of the vena cava in the right auricle (i.e., upstream from the coronary sinus), ScvO_2_ does not include myocardial oxygenation. On the contrary, SvO_2_ concerns venous blood from pulmonary artery, that is, by definition, after the coronary venous sinus. Such a difference might highly impact the observed values, given that (1) venous blood from the coronary sinus, with a saturation of oxygen close to 40%, is the most deoxygenated venous blood of the body [9] and (2) that in critically ill patients, myocardial oxygen supply/demand imbalance is likely to occur.

## 4. ScvO_**2**_: A Validated Monitoring Parameter

### 4.1. Experimental Validation

Many studies have compared the ScvO_2_ and SvO_2_ values in the same patients (Table 1). Most of them showed a good correlation between ScvO_2_ and SvO_2_ and a similar trend in the temporal evolution. In 1989, Reinhart et al. [10] reported, in a dog model, a correlation coefficient between ScvO_2_ and SvO_2 _of 0.96. In this study, the two values exhibited less than 5% difference in 77% of the cases. Later on, Reinhart et al. [11] confirmed their results in ICU patients: ScvO_2_ and SvO_2_ had similar evolution in 90% of the cases and had a correlation coefficient of 0.81 (*P *< 0.001). Similarly, Martin et al. [12] reported a parallel evolution of ScvO_2_ and SvO_2_ in 75% of the cases. Considering such results, it seems that ScvO_2_ and especially its evolution over time could be used as an interesting surrogate for SvO_2_ monitoring. However, the impact of ScvO_2_ monitoring on the prognosis of critically ill patients remained to be demonstrated.

### 4.2. Clinical Validation

Some authors, therefore, focused on evaluating the connection between SvcO_2_ and prognosis and especially the benefits turnoff considering SvcO_2_ optimization as a goal for resuscitation. Pearse et al. [23] observed in a cohort of 118 postoperative patients from major surgery that a decrease in ScvO_2_ during the first 8 hours was associated with an increase in 28-day morbidity and mortality. Consistently, Futier et al. [24] showed in major abdominal surgery that a ScvO_2_ <70% was associated with postoperative complications. In addition, ScvO_2_ seems to be a reliable and sensitive parameter to detect hemorrhage in trauma patients admitted to the Emergency Room [25], while other series suggest that ScvO_2_ could be a prognosis marker in myocardial infarction [26], acute heart failure [27], as well as in severe sepsis patients [28]. 

But the great clinical advantage related to early ScvO_2 _has been suggested by Rivers et al. [29]. Indeed, these authors reported that, in severe sepsis patients, an early and aggressive therapy that aimed at normalizing in the first hours the values of ScvO_2_ MAP and CVP achieved a reduction in in-hospital mortality from 46.5% to 30.5% (relative risk 0.58 (0.38–0.87), *P* = 0.009). These results were later confirmed by two large studies [30, 31] conducted, respectively, on 15,022 and 330 patients that both showed a mortality reduction related to the implementation of ScvO_2_ as a resuscitation goal. Though Levy's et al. study [30] failed to show any survival improvement specifically related to ScvO_2_ implementation, the global target implementation did (lactate measure, blood culture before antibiotics, broad spectrum antibiotics, fluid and vasopressors, CVP >8 mmHg, and ScvO_2_ >70%). This could be partly explained by the fact that, among those 6 resuscitation targets, ScvO_2_ >70% was the less commonly achieved, both after the first quarter of patients was included and after the final quarter of patients was included (resp., in 13.3% and 24.3% of the cases). Recently, Jones et al. [32] showed, in 300 septic shock patients, that the mortality of patients who benefited from a ScvO_2_ goal-directed therapy was low (23% (17–30%)) and similar to those who were treated using a lactate clearance goal-directed therapy (17% (11–24%)).

ScvO_2_ is considered as a suitable prognosis factor in many clinical situations in the critically ill patients. The Surviving Sepsis Campaign [33], gathering all European guidelines regarding severe sepsis and sepsis shock patients management, suggested including ScvO_2_ as a goal parameter in the first 6 hours of management (ScvO_2_ >70%).

## 5. ScvO_**2**_ Limits

### 5.1. Theoretical Limits

The first limit of using ScvO_2_ refers to its ignorance of the coronary sinus venous blood saturation. As the extremity of the ScvO_2_ catheter usually stands upstream from the joining point of coronary sinus in the right auricle, the ScvO_2_ value does not take into account the myocardial oxygen supply/demand adequacy. As myocardial oxygen extraction is physiologically basically high, venous coronary blood is one of the most deoxygenated venous bloods [9] of the body. This explains that the value of mixed venous blood saturation of oxygen (SvO_2_), which actually takes into account venous coronary blood, is usually lower than the ScvO_2_. Moreover, any major increase in myocardial oxygen consumption could lead to a critical myocardial oxygen extraction that would have no impact on ScvO_2_ monitoring. Besides, ScvO_2_, just as SvO_2_, is a global oxygenation parameter. So any local change in tissue oxygenation is at risk of being “diluted” in the rest of venous blood and then becoming undetectable. Similarly, in the case of a drop in regional venous saturation responsible for a drop of ScvO_2_, it would not be possible to assess the affected territory without further exploration. Then, theoretically, the distal extremity of the central venous catheter is supposed to be placed at the joining point of vena cava and the right auricle to enable a suitable assessment of tissue oxygenation of inferior and superior territories. However, checking the position of the catheter's distal extremity with chest X-ray is not accurate enough. Moreover, as venous saturation from the superior vena cava is systematically lower than inferior vena cava, any variation in the position of the catheter's tip could have a major influence on the measures and therefore lead to ScvO_2_ misinterpretation. Ultimately, as previously reported, ScvO_2_ depends on tissue oxygen extraction and hemoglobin affinity for oxygen. Experiments report that septic patients could suffer from a decrease in oxygen extraction capacity [34, 35], a rise in capillary shunt [34], as well as changes in hemoglobin affinity for oxygen [36]. All these changes may alter the theoretical relationship between SvcO_2_, and cardiac output, such as ScvO_2_ interpretation, to guide hemodynamic therapy becomes more complex.

### 5.2. Clinical Limits

First of all, one could argue that ScvO_2_ measurement requires a central venous catheter, which is an invasive technique, exposing patients to complications such as infection or hemorrhage. However, central venous lines are often needed for critically patients and could therefore be used for ScvO_2_ monitoring. However, in severe sepsis and septic shock, tissue hypoperfusion should lead to particularly low ScvO_2_ values, as observed by Rivers et al. in the early stage of sepsis. However, after the first hours of resuscitation, this situation is rarely met [37], and ScvO_2_ values tend to be paradoxically normal or even raised. This could be explained by the physiological modification induced by sepsis and previously described (decrease in tissue oxygen extraction capacity, rise of capillary shunt, and changes in hemoglobin affinity for oxygen). Consistently, in such situations, the agreement between SvO_2_ and ScvO_2_ seems much less satisfactory, especially in the context of septic shock [22, 38, 39]. Besides, ScvO_2_ clinical validation is mainly based on one single study [29], which is a single centre study, and its results are still controversial. As a matter of fact, van Beest et al. [37], in a Dutch prospective multicenter study, reported that only 1% of the patients meeting the inclusion criteria required by Rivers et al. [29] had a ScvO_2_ <50%. Ho et al. [40], in a retrospective study, as well as the ARISE group (Australian Resuscitation of Sepsis Evaluation), in a multicenter study [41], reported an in-hospital mortality of 26–28% in patients who did not benefit from an early goal-directed therapy but that met the inclusion criteria for Rivers' trial. This mortality rate is much lower than the one observed by Rivers in his control group. Finally, the low CVP values (5-6 mmHg) observed by Rivers et al. suggest that their patients were probably highly hypovolemic.

### 5.3. Global versus Regional Circulation

If global hemodynamic optimization is considered as an essential prerequisite to ensure adequate tissue perfusion, it may not be always sufficient to avoid the development of organ failure. The poor accuracy for global oxygen venous saturation monitoring to detect changes in regional oxygenation has been well described in animal models [42–44]. For instance, Legrand et al. [42] recently showed in a rat model that LPS-induced endotoxemia could induce alterations in microvascular perfusion and oxygenation in the renal cortex in rats, which appeared to be only weakly dependent on systemic and renal macrohemodynamic alterations. Consistently, Vallet et al. [44] and Lagoa et al. [43] reported, in endotoxemic dogs, that after resuscitation skeletal VO_2_ is maintained when blood flow within the gut is significantly disturbed with mucosal hypoxia. In human beings, as described by Sakr et al. [4]. global hemodynamic parameters fail to discriminate survivors from nonsurvivors after 24 hours of intensive care in septic shock patients. One illustrative example is the lack of accuracy of global SvO_2_ to detect cerebral venous desaturations [45]. In this perspective, global ScvO_2_ might face some limitations with respect to local inadequacy in the DO_2_/VO_2_ balance. Indeed, local SvO_2_ might not be detected by global oxygen saturation monitoring, the signal being *diluted* among a global normally saturated venous blood. Therefore, regional SvO_2_ could be an interesting supplementary target parameter. However, while regional SVO_2_ monitoring might be feasible at the bedside for some organ, such as jugular venous oxygen monitoring [46–48], it is much more difficult for others such as the kidney or the gut, for example. In such situation, some alternative parameters for regional monitoring could be of interest.

## 6. Candidate Parameter to Reflect Regional Inadequate Oxygen Supply

As for now, no biological or technical parameter has been proved to directly reflect regional oxygen supply inadequacy. Nevertheless, some parameters appear to be good surrogate candidate, such as tissue oxygen saturation (StO_2_) and regional carbon dioxide partial pressure (pCO_2_). 

### 6.1. Tissue Oxygen Saturation (StO_2_)

StO_2_ can be estimated by near-infrared spectroscopy (NIRS) using the differential absorption properties of oxygenated and deoxygenated hemoglobin. Near-infrared light (wave length 680–800 nm) easily crosses biological tissue and is only absorbed by hemoglobin, myoglobin, and oxidized cytochrome, but the contribution of myoglobin and oxidized cytochrome in light absorption is very low [49, 50]. Light tissue penetration is dependant on the space between the illumination fiber and the detection fiber. With a 25 mm space, 95% of the light signals detected come from a 0–23 mm depth.

The steady StO_2_ value is a reflection of oxygen saturation of the haemoglobin present in the tissue volume crossed by the near-infrared light, containing a mix of arteriolar, capillary, and venous blood. It is then a complicated integrative parameter, but it has been shown to be correlated to the microcirculation state and is therefore considered as an acceptable parameter for tissue perfusion [51]. 

During shock from various origins, the relationship between StO_2_ values at the forearm and the prognosis has been extensively studied during the past decade [52–55]. During shock states, as StO_2_ drops correlate with fall in central venous, mixed venous oxygen saturation, or oxygen delivery [56–59], StO_2_ seems to be a good marker of regional DO_2_/VO_2_ imbalance, with the advantage of being applicable to different regional territories such as the brain [60], the liver [61], or the muscle [62], for example. However, this technique suffers some limitations, the major one being its poor sensitivity to rule out tissue hypoperfusion [55]. In order to improve its sensitivity, vascular occlusion tests (transient upper arm arterial occlusion with a pneumatic cuff) have been proposed [63]. By continuously monitoring StO_2_ during the test, a pattern curve is obtained with an initial decrease of StO_2_ during occlusion, followed after cuff deflation by an increase of StO_2_ usually transiently reaching higher values than baseline (hyperemic response) before returning to baseline. The slope of the decreasing part of the curve is the StO_2_ desaturation rate and is correlated to the tissue oxygen consumption, whereas the slope of the increasing part of the curve is the StO_2_ recovery rate and is correlated to the quality of the microvascular bedside [64]. 

### 6.2. Carbon Dioxide Partial Pressure

Regional capnography relies on the principle that cellular oxygen consumption through oxidative phosphorylation produces proportional amount of carbon dioxide. In this perspective, any decrease in blood flow would result in a CO_2_ accumulation detected by a capnograph. Tissue pCO_2_ could then be used as a surrogate for regional blood flow and oxygen consumption combined [65]. However, regional pCO_2 _is difficult to interpret, because CO_2_ production also depends on cellular metabolism level and arterial glucose concentration. This probably explains the fact that, despite appealing, this parameter is still rarely used in clinical practice.

## 7. Conclusion

In conclusion, ScvO_2_ measurement seems to be an interesting tool, especially in the early phase of shock to guide fluid management and blood transfusion or inotropic support. Nevertheless, a large knowledge of its determinants and the physiology of circulation seem to be essential to ensure a reliable interpretation in clinical practice. When ScvO_2_ is low, it reflects an adaptive mechanism to an unsuitable supply in oxygen and should lead doctors to understand the reasons for it and to propose an appropriate optimization strategy. As well, in clinical situations such as septic shock, after the first hours of management, a “normal” or even a high ScvO_2_ can be falsely reassuring.

## Figures and Tables

**Table 1:** Summary of the studies comparing SvO_2_ and ScvO_2_ in humans or in experimental models.

Author (year)	Type of patients (*n*)	Conclusion	Correlation coefficient
Tahvanainen et al. [13] (1982)	Intensive care (42)	ScvO_2_ = SvO_2_	NC
Wendt et al. [14] (1990)	Intensive care (19)	ScvO_2_ ~ SvO_2_	0,78
Kong et al. [15] (1990)	Kidney failure (8)	ScvO_2_ ~ SvO_2_	NC
Berridge et al. [16] (1992)	Intensive care (51)	ScvO_2_ = SvO_2_	0,92
Herrera et al. [17] (1993)	Thoracic surgery (23)	ScvO_2_ = SvO_2_	NC
Pieri et al. [18] (1995)	Major surgery (39)	ScvO_2_ *≠* SvO_2_, nonsubstituable	0,90
Ladakis et al. [19] (2001)	Intensive care (61)	ScvO_2_ = SvO_2_	0,94
Reinhart et al. [11] (2004)	Intensive care (32)	ScvO_2_ ~ SvO_2_	0,81
Chawla et al. [20] (2004)	Intensive care (53)	ScvO_2_ > SvO_2_	0.88
Dueck et al. [21] (2005)	Neurosurgery (70)	ScvO_2_ *≠* SvO_2_, substituable evolution	≥0,75
Ho et al. [22] (2010)	Intensive care	ScVO_2_ *≠* SvO_2_, nonsubstituable	NC
